# A Critical History of Co‐Production in the Design and Delivery of UK Mental Health Services

**DOI:** 10.1111/hex.70865

**Published:** 2026-09-17

**Authors:** Pete Fleischmann, Peter Schofield

**Affiliations:** ^1^ Independent Consultant (Co‐production Works); ^2^ Department of Population Health Sciences King's College London London UK

**Keywords:** co‐production, history, mental health, mental health services

## Abstract

**Background:**

The term co‐production is now very common and risks losing its original egalitarian and emancipatory meaning. Questions about what the term means relate to how the concept developed historically. We conduct a critical history of the concept to shed some light on this and inform contemporary practice.

**Objectives:**

To chart the history of co‐production focusing on co‐production in the design and delivery of mental health services; critically examining the extent to which the original egalitarian promise has been realised.

**Methods:**

We draw on early foundational texts, key UK policy frameworks and legislation, mental health service user/survivor movement accounts and more recent guidelines, using illustrative cases to critically examine the concept.

**Results:**

Early accounts from the US stressing reciprocity, shared power, and active citizenship directly influenced approaches to UK mental health services. Further accounts from the user/survivor movement stressed the importance of power sharing and a critique of medical models of mental distress. UK policy frameworks and legislation have since brought co‐production from the margins to the mainstream, introducing a tension between a mainstream concept of co‐production influencing services through collaboration and a service user/survivor concept of co‐production as equal power sharing.

**Conclusion:**

Historically there have been a range of conceptions of co‐production with different notions of what power sharing means for service users working alongside professionals.

**Lived Experience or Public Contribution:**

Both authors have past lived experience of using psychiatric services. The manuscript was also shared with a small group of user/survivors with a particular interest in co‐production and their feedback incorporated in the final draft.

## 1 Introduction

The term co‐production is now commonplace in discussions of user involvement and, it is argued [1, 2], risks being overused and diluted to the point where its original meaning becomes lost. Co‐production's original egalitarian and emancipatory promise has a special importance for mental health services for a number of reasons. Firstly, the notion of co‐production as the equal sharing of power between professionals and service users is a particular challenge in mental health given its uniquely asymmetrical balance of power. As one recent review shows, just under one third of people diagnosed with first episode psychosis are involuntarily admitted to hospital at first presentation [3]. As the one medical discipline with a statutory framework for coercive treatment for those who retain capacity, psychiatry is arguably poorly placed to promote power sharing [4]. Furthermore, the notion of mental health service users as equal knowledge partners inevitably comes into conflict with a medical model that can undermine their testimony as irrational, symptomatic and therefore untrustworthy [5]. At the same time notions of scientific objectivity and professional distance may sit uneasily alongside service users' personal experience of mental distress [2]. This, coupled with the enduring stigma associated with mental illness, makes the role of mental health service users as equal partners a significant, and some argue [4], almost impossible challenge. At the same time, the enthusiasm with which mental health services have embraced the concept of co‐production should be seen in the context of a historically recurring crisis of legitimacy experienced by the mental health professions [6]. Beset with controversy over diagnosis [7], appropriate treatment [8] and the management of risk [9], mental health services have arguably needed co‐production as much as their recipients.

While the notion of co‐production in mental health work has significant challenges, the concept itself has proved slippery, often meaning quite different things to the different actors engaged in notionally co‐produced work. It is argued [1, 10, 11] that this lack of conceptual clarity is a fundamental problem which risks undermining the efforts of mental health service users who simply believe co‐production means that they will have an equal say. While there are many examples of this in recent years, we highlight one particularly striking example. Oxevision provides 24‐h video surveillance in the bedrooms of acute psychiatric inpatients. This has proved to be consistently controversial among both service user groups and mental health advocacy organisations to become one of the most controversial mental health initiatives of recent years [12]. However, Oxehealth (now rebranded as LIO), who make Oxevision, confidently claim to be expert in co‐production [10, 13], extolling the benefits of applying a ‘co‐production approach’ throughout their work. Although their website is vague about exactly how, in practice this appears largely confined to co‐produced patient information (e.g. leaflets and posters) and guidelines along with some involvement in research [14, 15, 16]. However, as one review concludes, the latter has been deemed low quality due to their own conflict of interest [17]. We highlight this as probably the most audacious example of how co‐production can be interpreted differently, although this is not the only unpopular initiative to align itself with co‐production in this way [18, 19, 20].

So how have we reached this point? The potential for very different interpretations of co‐production arguably goes back to the way the concept developed historically. Accounts often differ in the sources they attribute to the concept's origin, typically according to discipline [21], and definitions themselves vary widely. Given the challenges outlined above, we therefore propose a critical approach to the history of co‐production applied to the design and delivery of mental health services, to shed some light on these contemporary issues. By critical history we mean the process of examining often taken for granted concepts to explore how our understanding of their meaning has evolved over time; focussing, in particular, on the power relations at work in this process. We examine the key components of the concept, and what has made this distinctive from simply involving users of services, as well as some potential conflicts that may be inherent in the way co‐production is defined. While highlighting the contribution of the mental health service user/survivor movement, our account also looks at academic and policy level contributions, and the tensions between all three.

Defining co‐production is itself a challenge as there is no fixed universally agreed definition, and this is further complicated where activity definable as co‐production is often not described as such by those involved or in the literature. The historical account that follows therefore interweaves the various definitions given over time. We look specifically at how the concept of co‐production has developed in the UK; drawing on those sources that have been most influential, both internationally and from within the UK. While we present concepts and definitions that are widely shared, we acknowledge that this reflects our own perspective as users/survivors working in mental health. However, writing from this perspective we argue that our historical account, situated in this context, provides an important addition to the literature on co‐production.

The following account begins by looking at the origins of the concept; how this came to be adopted in the UK and by UK mental health services; the role played by the mental health service/user survivor movement; and lastly, how co‐production entered the mainstream through UK policy and legislation.

### 1.1 A Note on Terminology

There are many terms used to describe people with direct experience of using mental health services or with experience of mental ill‐health, distress or trauma who engage with professionals in co‐production activities. These include experts by experience, people with lived experience, service users and survivors. In this paper we use the terminology favoured by the National Survivor/User Network (NSUN). They use the term people with lived experience of mental ill‐health, distress or trauma and also the terms user/survivor. In this paper we use a range of short forms such as service user, people with lived experience typically reflecting the terminology used by the authors we are discussing.

### 1.2 The Origins of Co‐Production

We begin by examining the origins of the term co‐production. It is in the stories of two Americans, Elinor Ostrom an academic economist and Edgar Cahn, a civil rights lawyer, that we find the original governing narratives of co‐production [22, 23]. It is commonly agreed [24] that Ostrom first used the term co‐production in 1973, in the context of public services, to explain why crime rates increased in Chicago as police work shifted from foot patrols to cars [25]. The argument being that the relation between police and community was essentially reciprocal. She describes co‐production as:

…the process through which inputs used to produce a good or service are contributed by individuals who are not in the same organisation. [26]

This has become a common definition of co‐production although this is sometimes misused to imply that co‐production is happening when, for example, a local authority and an integrated care board work together. However, later in the same paper, Ostrom clarifies this in terms of the potential relationships between:

‘regular producers’ (police officers, teachers, health workers) and ‘clients’ who want to be transformed by the service into safer, better educated or healthier persons. [26]

with the term client in inverted commas as Ostrom considered this a passive term, i.e. that clients are acted upon. However, in her view co‐production meant citizens could play an ‘active role in producing public goods or services of consequence to them’ [26]. Though Ostrom's work is seen by some as more limited and less radical than what was to follow [27, 28], she began describing the process of empowering citizens and providing opportunities for their active participation in service development and delivery. Ostrom argued that, following the experience of co‐production, citizens develop confidence, building networks and often become activists in other areas.

Starting in the 1980s Edgar Cahn, a former staffer for Robert Kennedy, lawyer and anti‐poverty activist, developed and popularised his own brand of co‐production. Through Cahn's activities and his book, ‘No More Throw–Away People, The Co‐production Imperative’ [29], he created his own mythology. He relates how he was in hospital recovering from a heart attack, lying helpless in bed, not knowing if he would live or die. Through this experience of helplessness, he had an epiphany; with the realisation that most social and health systems are based on dependency relationships which was why they were often ineffective. From this he developed his theory of co‐production building upon Ostrom's ideas.

Cahn put his thinking into practice, developing a non–monetary exchange system he called ‘Time Dollars’ [30]. Time Dollars enabled people to exchange an hour of their time with an hour of someone else's. Everyone's time was valued equally and through the system of credits, people could exchange with anyone in the network. Time dollars, as he himself said, were only a tool; the real invention was his co‐production approach.

His theory of co‐production is built around the ‘Core Economy’. In contrast to the market, the Core Economy consists of the work and support provided by family, friends and community members which is not valued in monetary terms:

Family, neighbourhood, community are the Core Economy. (which) produces: love and caring, coming to each other's rescue, democracy and social justice. [24]

arguing that co‐production has three aspects: firstly, that the Core Economy has ‘parity with the world of money and market’. Secondly it is a *process*, which:

may be smooth and cooperative or it may take the form of a dialectic that yields parity, only after a struggle because the process entails a shift in status that may be embraced or resisted.

Lastly, it comprises a ‘set of standards or goals: an asset perspective, redefining work, reciprocity and social capital’ [29]. For Cahn, co‐production clusters around these overlapping concepts; an asset–based approach is about acknowledging that everyone has something to contribute. Reciprocity is about mutual benefit: ‘a two–way street is better than a one–way street’ [29].

Permeating this approach is an insistence that co‐production is about social justice. Co‐production is an imperative which demands ‘No more free rides for the market economy’ and ‘an end to devaluing people and profiting from their troubles’ with'no more disinvesting in families, neighbourhoods and communities’. [29]

While this clearly resonates with community‐oriented approaches to mental health, people with mental health problems appear infrequently in ‘No More Throw–Away People’. There is just one example, ‘the Story of George’, intended to illustrate the power of reciprocity. George is described as ‘a gentle soul’, ‘a schizophrenic with nearly 19 years of residential and outpatient treatment… at an institution for the mentally ill’ [29]. Unable to sustain employment, George finds using public transport and getting out of bed difficult and is happiest grooming horses. Cahn explains how in exchange for legal support, George was asked to help distribute food at a Time Dollar warehouse. He emphasises George's delight that he could be useful to his lawyer. George is ideal for his role at the food bank which involves hard manual labour, as he has the ‘build of a weight lifter’. While this story is presented as a fable of reciprocity George's role appears to be largely that of a grateful supplicant rather than equal partner.

‘No More Throw–Away People’ is a strange hybrid, part how–to guide, part thesis; it is also an auto–hagiography and a call to action, using stories, poetry and biblical quotations to exhort and inspire. Despite its contradictions, or perhaps because of them, ‘No More Throw–Away People’ and Cahn himself have had a major impact on the development of co‐production in the UK. In fact, some argue that the success of co‐production may in fact stem from the ambiguity and flexibility of the term allowing its use to be so easily adopted in such a wide range of applications [21]. As we have shown, the concept was developed outside of the mental health field and its emancipatory promise was not always realised when applied to mental health.

### 1.3 Development in the UK

In the late 1990s, the New Economics Foundation (NEF) think tank, supported by the National Endowment for Science, Technology and the Arts (NESTA), introduced this model of co‐production to the UK. NEF formed strong links with Edgar Cahn and were instrumental in setting up several Time Dollar services which they re–named Time Banks. The most well–known of these was established in 1998 by Rushey Green General Practice (GP) surgery [31] with one of its aims to address the social isolation of those diagnosed as depressed.

NEF further developed this approach and published, in 2008, a Co‐production Manifesto for which Cahn wrote the foreword [24]. In 2010 NEF then published, ‘Public Services Inside Out’, which took his four core values of co‐production, added two more and adapted them to the context of UK public services [32]. NEF presented these as the ingredients required by a co‐production project with each chapter focusing on a specific ingredient:

1. Building on people's existing capabilities
2. Mutuality and reciprocity
3. Peer support networks
4. Blurring distinctions
5. Facilitating rather than delivering
6. Recognising people as assets [32]

These principles are often quoted in the co‐production guides, frameworks and toolkits, for example Skills for Care [33], which have proliferated in recent years. Many organisations have also developed their own principles informed by NEF's original six, for example the Social Care Institute for Excellence [34], the Coalition for Collaborative Care [35] and the National Lottery Community Fund [36]. NEF developed a corresponding definition of co‐production as:

A relationship where professionals and citizens share power to plan and deliver support together, recognising that both have vital contributions to make in order to improve quality of life for people and communities. [37]

Through its publications and activities, NEF provided a theoretical framework for co‐production in mental health in the UK. However, despite NEF's calls for more equal relationships, reciprocity and the development of peer networks; their writing and practice largely ignored the burgeoning movement of people who use mental health services which had been gaining strength since the 1970s. The way that co‐production is evidenced in their publication ‘*Co–production: A manifesto for growing the core economy* ‘ [24] has a strange echo of Cahn's ‘Story of George’. They provide the example of Bee Harries, a local mental health service user, who received an individual budget and was also helped to become involved in a network through which she was part of a poetry writing group. However, Ms Harries is mostly a passive voice in the Manifesto, with only one short direct quote: 'our life does not have to be going from one drop–in centre to another'. The rest of the text frames Ms Harries' experience as an example of the benefits of co‐production, mutuality and reciprocity in language it is unlikely she would have used herself. Contrast this with the Manifesto's description of Zoe Reed, then Director of community services at the South London and Maudsley NHS Foundation Trust (SLaM), as one of ‘a growing band of prophets of co–production’. The other prophets listed are all senior professionals and academics.

One attempt to combine both Cahn and NEF approaches is the Social Care Institute for Excellence (SCIE) guide: ‘Co‐production in Social Care: What It Is and How to Do It’ [34]. The process of developing the guide was highly co–productive, including a user–led steering group and authors with lived experience of mental health issues. The guide attempts to combine policy, academic work and knowledge generated by people who use services. It proposes four co‐production values: Equality (power–sharing), Diversity, Accessibility and Reciprocity; drawing on both the Cahn/NEF models but also incorporating the concerns of the disability and user/survivor movements around power, accessibility and diversity. SCIE was one of the first social care organisations to champion co‐production, developing its own sophisticated internal co‐production structures and processes and disseminating guidance and good practice, through activities such as national co‐production week and their co‐production festival [37].

In this way the term was gradually adopted in UK mental health care, albeit with differing models and definitions.

### 1.4 The User/Survivor Movement and Co‐Production

In our experience co‐production is often presented as a process whereby a group of unconnected individuals are invited or recruited to work with professionals. However, as others have highlighted [38, 39], this is only one interpretation and early discussions of co‐production clearly outline how co‐production also works at a group and collective level [40]. The mental health user/survivor movement represents the collective interests of mental health service users and, we argue, has had a significant impact shaping co‐production. However, the organisations and individuals involved in the movement have only relatively recently described their work in this way. In the next section, we consider and contrast the user/survivor movement's contribution to shaping co‐production.

The service user/survivor movement can be described as comprising those

who speak out for their own rights and those of others, and local groups and national organisations set up to provide mutual support or to promote the rights of current and former mental health service users to have a voice. [42]

A key feature of the user/survivor movement's thinking is a critique of the biomedical model of mental illness along with a questioning of the imbalance of power between psychiatric professionals and service users. Despite decades of discussion and advocacy for more holistic ‘biopsychosocial’ models of mental distress, the reality for many service users is a continuing reliance on medication as the primary recourse to address often profound mental health problems [41]. By treating mental health problems as essentially brain diseases, and therefore under an exclusive medical remit, the medical model de‐prioritises the right to self‐determination, the importance of patients' individual life circumstances and social/psychological explanations [42, 43]. While this critique takes various forms in the user/survivor movement there are common concerns, including: calls for less medicalised and coercive services, alternatives to medication, greater availability of advocacy and an end to societal discrimination [44].

In the UK the modern user movement emerged in the 1970s with the formation of local Mental Patients Unions in Manchester, Portsmouth and the London borough of Hackney. Since its inception there has been a tension between those who believe the focus should be on developing user–controlled self–help services and those attempting to reform the current system through collaboration with professionals [1]. In 1978, Judi Chamberlin an American ‘ex‐mental patient’ published ‘On Our own’, setting out the case for patient–controlled services [45]. Chamberlin argued passionately for a patient–controlled alternative to biomedical psychiatry. She maintained that, because the power differentials between professionals and service users were so significant, collaboration would not bring about change. Similarly, ‘Stopovers on my way home from Mars’ [46] an influential study of the mental health user movement in the UK, Holland and the USA by a New Zealand user/survivor, Mary O'Hagan, is full of warnings of the dangers of collaboration:

When we fail to link our experience with our ideology and ideology with our practice we are no longer a powerful force for change. Instead we tend to parody the system that has dehumanised us. [47]

Collaboration in this sense meaning aligned with the priorities of professionals where, as Rose and Kalathil argue, the promise of equality can too easily become hollow where power asymmetries remain hidden and unresolved [4].

As Harcourt argues, while sharing similar goals, user‐controlled services are something quite distinct from co‐production because this ‘exceeds the aspiration of equalising the relationship’ [1]. However, beginning in the 1980s, the phrase user‐involvement began to be frequently used to describe collaboration between service users and professionals. Local user groups were funded, and in some cases set up by, local authorities with the explicit intention that they would become involved in the design and evaluation of services. Despite fitting well with most, if not all, of NEF and Cahn's typologies and Ostrom's model of ‘regular producers’ working with clients, it was only relatively recently that this type of activity became referred to as co‐production.

In the late 1980s the user movement entered a new phase of more significant organisation and effectiveness. In 1986 Survivors Speak Out (SSO), a radical network of user/survivors which organised events and publications, was founded by Peter Campbell. A user/survivor, Jan Wallcraft, became the first staff member at MINDlink, national Mind's (England & Wales charity) internal network of user/survivors, the result of a deliberate decision to employ a user/survivor coordinator. In 1992 the United Kingdom Advocacy Network (UKAN), a national umbrella organisation covering UK mental health service user groups, was set up with a membership of more than 100 local survivor groups.

During the 1990s there was increasing awareness of the over–representation of ethnic minority people in the mental health system and concerns that white people dominated the user movement [44]. Addressing this several black and ethnic minority user groups were formed. For example, in 1998 SIMBA (Share In Maudsley Black Action), the Black Patient/User/Survivor group, was established [47]. During the early 2000s other groups and organisations such as Catch‐a‐Fiya, and the Afiya Trust provided a platform for user/survivors from racialised groups to share knowledge and experiences. However, by 2016 SIMBA, Catch‐a‐Fiya and the Afiya Trust had all folded due to funding cuts. This demonstrates how difficult it is for both the user/survivor movement and the broader practice of co‐production to avoid echoing societal inequalities and deeply entrenched power relations [4].

During the early years of this century, the user/survivor movement continued to grow and develop. However, it was during this period that both SSO and UKAN struggled to secure funding and became less effective. In 2003, a review of the user/survivor movement was published ‘On Our Own Terms’ [44] articulating concerns about the effectiveness of service user involvement and arguing for more training and resources, for both professionals and users, to address this. It expressed particular concern around the importance of addressing power differentials between user/survivors and professionals including how users/survivors were valued and compensated for their involvement. The report also recommended setting up a national user/survivor network. In 2007, following a conference, Doing it Ourselves, the National Survivor User Network (NSUN), was set up with funding from two grant giving trusts [48]. NSUN trod a delicate path, which itself reflected the general development of the UK survivor movement. NSUN engaged with government funded projects such as the 4PI standards for involvement [49] but also remained an independent user‐controlled organisation which campaigned on issues such as the review of the mental health act. By 2017, NSUN had 4,500 individual members and was in touch with 100 s of user–led organisations. However, the years of government austerity from 2008 were detrimental to the user movement. In 2019, NSUN estimated that 200 user–led groups had closed over the previous 2 years [50].

The expansion of the user movement therefore played an important role in the development of co‐production in the UK. However, this has not always been acknowledged by other key stakeholders such as academics, think‐tanks and mainstream charities. See, for example, the literature review conducted by the New Economics Foundation and commissioned by National MIND which makes no mention of the user/survivor movement contribution to shaping co‐production [51]. This may partly reflect the issue that user/survivor groups are often poorly funded and less able to promote their ideas. Therefore, knowledge about the user movement often appears as ‘grey’ or informally published literature rather than appearing in more reputable and academic sources [4]. It may also reflect the separatist current that still runs through some local and national organisations. Another important factor is terminology because, until relatively recently, users and survivors did not call their activities co‐production. For example, when in 2013, NSUN originally developed their ‘4PI National standards for the meaningful involvement of people who use mental health services, their family and carers’, the original documents did not use the word co‐production [52]. However, 4PI is now referred to on NSUN's website as being about ‘ensuring effective co‐production’ [53].

Perhaps the most valuable contribution the user/survivor movement has made to the development of co‐production in the UK is a realistic understanding of the efforts that need to be made to equalise power differentials if co‐production is to be meaningful. Sarah Carr, a user/survivor researcher and former Co–Chair of NSUN, develops the argument that a collaborative relationship requires*. ‘close consideration of the fundamental power realignment, preparation, facilitated personal reflection and individual role renegotiation that needs to occur before this is possible’.* [27]

So, a number of questions remain about the impact of user involvement and/or co‐production. Is it possible for truly equal and reciprocal relationships to develop between user/survivors and professionals especially given the continuing reliance on biomedical, i.e. medication based, models of mental distress? Does the practice of co‐production simply further disguise rather than reveal the reality of unequal power relations? Without a coherent critique of the prevailing medical model and strong roots in the user movement how can individual service users/survivors collaborate with professionals as genuinely equal partners? Herein lies a potential fault line between the NEF/Cahn model of co‐production and the mental health user/survivor movement. While the movement is diverse and some members broadly accept a biomedical viewpoint, there is a consistent critique of many aspects of the medical model and the slow emergence of a fundamentally different approach to mental health based around an understanding of the impact of trauma and structural inequalities and a desire for self‐determination and emancipation. Despite assertions about co‐production needing to be linked to social justice, a specific critique of the mental health system is lacking from NEF's account. NEF seems to be arguing that if everyone worked together services would improve and that this can happen without any real change in the ideology, power relations or culture of the system.

To summarise, the user/survivor movement has played an important role in the development of co‐production in UK mental health, emphasising the importance of equal‐power sharing and a critique of the bio‐medical model as opposed to simply collaborating in service development.

### 1.5 Legislation and Policy Supporting Co‐Production

Citizen participation in health and social care services can be seen as a phase in the development of democracy and human rights [54] User involvement and co‐production policy in this context can be seen as part of a historical continuum which starts with the development of universal suffrage and fundamental rights. However, the promotion by the state of greater citizen participation has occurred during a period of neo–liberal dominance of social policy. As a result, progress around user involvement/co‐production has coincided with cutbacks in public services and welfare provision. So whilst ideas and discourses around citizen rights and participation have grown, at the same time some basic services have been withdrawn and certain fundamental liberties curtailed [55].

Since 1961, when Health Minister Enoch Powell made his famous Water Tower Speech calling for the dismantling of the large asylums, UK mental health policy and legislation has steadily given greater emphasis to the rights of people who use mental health services including the understanding that they have a role to play in the design of services. This gathered pace with the National Health Service and Community Care Act (1990), which began the development of Care in the Community and also made consultation with service users and carers a legislative duty for local authorities. In 1991, the Mental Illness Specific Grant (MISG) was introduced [56] which allowed local authorities to support local mental health user groups. As a consequence, by 1992 more than a hundred local user/survivor groups existed across the UK [47].

In 1992, the Mental Health Task Force Service User Group (part of Department of Health's Mental Health Task Force) was set up, initiating a series of regional service user conferences and publications, including guidelines for service user charters and advocacy. Although not described as such, this was an exemplar of co‐production with people who use services, professionals and policymakers working together. Representatives of the three main working groups; MINDlink, SSO and UKAN sat on the national Task Force and organised regional events [47]. In 1999, the Department of Health published ‘A National Service Framework for Mental Health’ which committed to involving users and carers including from Black and ethnic minority groups in service development, monitoring, and staff training [57].

From 1999 to 2007, policy and legislation continued to support the involvement of people who use services, their family carers and the general public. In 2000, *The NHS Plan* included a requirement for Patient and Public Involvement Forums to be set up in every NHS trust and primary care trust in England [58]. The aim was for these forums to allow local people an active role in decision making. Despite policy setbacks to the growth of service user involvement, such as the 2001 abolition of Community Health Councils (which had often served as incubators for local user groups), the user/survivor movement continued to grow and have an impact on services.

The review of the 1983 Mental Health Act, which started in 1999, resulted in the introduction of Community Treatment Orders in 2007. Perceived as a major defeat for the survivor movement this extended statutory powers to force people to adhere to treatment regimes after their discharge from hospital. However, more positively, the 2007 review included restrictions on the use of electroconvulsive therapy and introduced statutory rights to independent mental health advocacy. Despite these progressive measures the outcome of the review demonstrates the limitations of the influence of people with mental health issues on policy at the highest level.

Significantly in 2007, co‐production was first mentioned in government policy. The *Putting People First* concordat proposed that the transformation of adult social care services toward a more personalised approach would ‘be the first public service reform programme which is co–produced, co–developed, co–evaluated’ and that ‘real change will only be achieved through the participation of users and carers at every stage.’ [59]

*Putting People First* policymakers recognised that, to co‐produce, service users and carers would need their own organisations to develop a collective vision for local services and co–ordinate individual contributions. Local authorities were strongly encouraged to ensure that they developed and sustained User–Led Organisations. As the Department of Health's ‘Local Authority Circular No.1’ [60] stated: ‘Where user led organisations do not exist, a strategy to foster, stimulate and develop these locally should be developed’. Also, a programme to support and strengthen User–Led Organisations was set up at the Office for Disability Issues [61].

In 2009, the Cabinet Office published ‘Co‐production in Public Services, A New Partnership with Citizens’. This discussion paper defined co‐production as ‘a partnership between citizens and public services to achieve a valued outcome’ [62]. It argued that co‐production should be central to the improvement of all public services, outlining the changes needed to support co‐production. These included: control of budgets being given to service users and front–line staff and backing for peer support and changes to professional training and culture.

The statutory guidance of the Care Act 2014 was the first time that co‐production was defined in legislation:

‘Co‐production’ is when an individual influences the support and services received, or when groups of people get together to influence the way that services are designed, commissioned and delivered. [63]

This definition is seen by some commentators as weak, mainly because of the use of the word ‘influence’ [34]. However, the guidance does recommend that co‐production should be used to develop local implementation strategies, information and advice plans, to design preventative approaches, conduct individual assessments, shape markets and create mutual support networks.

The *5 Year Forward View* for mental health published in 2016 is more explicit; arguing for embedding co‐production ‘at the heart of commissioning and service design’ and ‘working in partnership with voluntary and community sector organisations’ [64].

The emphasis on co‐production in government policy shows little sign of abating. For example, the 2022 Health and Care Act includes legal duties for Integrated Care Boards to involve the public and work with communities. Furthermore, NHS England's 2024 Commissioning Framework for Mental Health Inpatient Services is clear that ‘New models of care and support should be co‐designed with people with recent lived experience of inpatient provision’. In this way co‐production continues to move from the margins to the mainstream of mental health care and support.

Most recently HealthWatch England has been the key national government agency supporting public involvement in Health and Social Care services. While HealthWatch England has not taken a clear position on Co‐production, several local HealthWatches, have been strong supporters. However, at the time of writing HealthWatch is at risk of abolition which could indicate a waning of interest in co‐production by the current administration. But it is interesting to also note that, following the defeat in parliament of proposals for the reform of welfare benefits, the government review of Personal Independence Payments (PIP) is being co‐chaired by disabled people. This is described as ‘the largest co‐produced review ever undertaken by government at a national level’.

The term co‐production has therefore moved to the mainstream supported by national policy initiatives but, in some cases, with its original promise diluted. However, as we have shown, this remains a complex and ever‐changing field.

## 2 Discussion

A number of themes are apparent from this overview of the history of co‐production as applied to mental health: firstly, the tension between the egalitarian ideals of co‐production and their practical realisation. From the beginning the notion of equal partnership has conflicted with the way this is discussed with reference to mental health service users themselves. As we highlight, this is seen in Cahn's account of the ‘schizophrenic’ whose agency is limited to what professionals are prepared to concede, and the example of Bee Harries, a similarly passive recipient of co‐production in NEF's account. The way the egalitarian promise of co‐production can be easily distorted may in turn reflect the historically very broad definition of the concept. With its roots in public administration, co‐production could be simply interpreted as a more efficient way of developing services by incorporating the views of consumers. This is reflected in those contemporary accounts that follow a technocratic rationale: i.e. co‐production is more efficient; rather than a democratic rationale: i.e. co‐production is more egalitarian [2]. While early accounts distinguish between the different levels at which co‐production operates, i.e. individual versus group or collective levels, the former often dominates contemporary accounts. An important corrective to this is the role of the mental health user movement. This has been key to shaping and formulating an emancipatory definition of co‐production that goes beyond simply making services more efficient and ‘user friendly’. Whether co‐production occurs at an individual or group level then becomes key. At an individual level it is hard to see how power dynamics could operate equally given that mental health professionals are, by definition, working as members of a group with all the status and privilege implicit in membership of that group. While service user participants' role might be to represent a ‘patient voice’, they themselves do not benefit from a collective identity in the same way as professionals. Mental health service user groups involved in co‐production can help fill this gap and it is here that we see much of the effort in recent years to formulate effective co‐production strategies, for example, Capital user/survivor group in west Sussex [65].

A further theme in our account of the user/survivor movement's role concerns ideology: the importance of a critique of reductive medical models along with a rebalancing of power in favour of the priorities of users themselves. There is though still a tension between those who advocate for user *led* services and those who advocate for co‐production. Co‐production as collaboration can be seen to act against the interests of people with lived experience where their interests are not aligned with professionals. A consistent feature of definitions of co‐production coming from the user movement is therefore that this must involve professionals ceding power in some meaningful way, such that co‐production is likely to work against the mainstream [2, 4, 10].

Our account has some limitations which we acknowledge. Firstly, while we begin with the origins of the term in the US, our account of recent co‐production history is largely written from a UK perspective because this is the area we are most familiar with. We recognise that the way that the term is used is highly contextual and therefore this is one of a number of parallel histories that could be written. Secondly, we have largely confined our account to co‐production where it directly concerns mental health services rather than co‐production in academic research. While there are many overlapping issues, the differing objectives and priorities of research warrant a separate account.

As we demonstrate in this paper the history of co‐production delineates the steady acceptance of the idea that the citizen has a role in improving mental health services. However, government and the mental health sector struggle to turn policy aspirations into reality: services that truly foster equal and reciprocal relationships between those who use services and professionals. The history of co‐production is also the history of the user/survivor movement which reimagines mental health services according to a social model. As we argue here, it is only by insisting that co‐production itself must be co–produced and co–owned by user/survivors, that the promise of co‐production can be fulfilled.

## Author Contributions

**Pete Fleischmann:** conceptualisation, investigation, writing – original draft, methodology, validation, writing – review and editing, supervision, project administration. **Peter Schofield:** writing – review and editing, conceptualisation, project administration, supervision, methodology, validation, investigation.

## Ethics Statement

The authors have nothing to report.

## Conflicts of Interest

The authors declare no conflicts of interest.

## Data Availability

The data that support the findings of this study are available from the corresponding author upon reasonable request.
